# PPDMs—a resource for mapping small molecule bioactivities from ChEMBL to Pfam-A protein domains

**DOI:** 10.1093/bioinformatics/btu711

**Published:** 2014-10-26

**Authors:** Felix A. Kruger, Anna Gaulton, Michal Nowotka, John P. Overington

**Affiliations:** ChEMBL group, EMBL-EBI, Wellcome Trust Genome Campus, Hinxton, UK

## Abstract

**Summary:** PPDMs is a resource that maps small molecule bioactivities to protein domains from the Pfam-A collection of protein families. Small molecule bioactivities mapped to protein domains add important precision to approaches that use protein sequence searches alignments to assist applications in computational drug discovery and systems and chemical biology. We have previously proposed a mapping heuristic for a subset of bioactivities stored in ChEMBL with the Pfam-A domain most likely to mediate small molecule binding. We have since refined this mapping using a manual procedure. Here, we present a resource that provides up-to-date mappings and the possibility to review assigned mappings as well as to participate in their assignment and curation. We also describe how mappings provided through the PPDMs resource are made accessible through the main schema of the ChEMBL database.

**Availability and implementation:** The PPDMs resource and curation interface is available at https://www.ebi.ac.uk/chembl/research/ppdms/pfam_maps. The source-code for PPDMs is available under the Apache license at https://github.com/chembl/pfam_maps. Source code is available at https://github.com/chembl/pfam_map_loader to demonstrate the integration process with the main schema of ChEMBL.

**Contact:** jpo@ebi.ac.uk

## 1 Introduction

Systematic analyses of bioactive small molecules and their molecular targets and homologues form the basis of a number of novel applications in computational drug discovery and systems and chemical biology, including methods of target prediction (Martínez-Jiménez *et al.*, 2013), and for the establishment of functional relationships between proteins (Kruger and Overington, 2012; Lin *et al.,* 2013; van der Horst *et al.,* 2010). To add precision to these methods, we have previously proposed a simple mapping heuristic of small molecule bioactivities to protein domains (Kruger *et al.*, 2012). Here, we present a full implementation of the mapping to a relevant subset of biological assays stored in the current version of the ChEMBL database (Bento *et al.,* 2014). This implementation also accommodates edge cases that were unaddressed in the original implementation—specifically cases where more than one Pfam-A domain could mediate small molecule binding. In the refined implementation, such cases were resolved manually. The PPDMs server provides a platform to review and contribute manual assignments.

Previous computational approaches exist that link small molecule binding to specific protein domains, but these approaches use information extracted from crystal structures of protein-ligand complexes to transfer binding annotations between homologous proteins (Davis and Šali, 2010; Finn *et al*., 2014; Snyder *et al*., 2006). More recently, such approaches have been used to associate protein domains based on ligands shared between them (Li *et al*., 2012; Moya-García and Ranea, 2013). PPDMs provides an alternative approach that associates small molecule binding and protein domains based on empirical evidence from literature reported measurements in biological assays. PPDMs has generated binding-domain annotations for ∼770 k small molecule bioactivities which can be obtained from the main schema of the ChEMBL database.

## 2 PPDMs enables improved mapping of ligand-binding domains

**Fig. 1.**
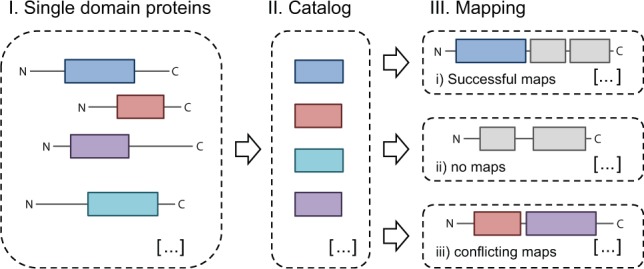
Schematic illustration of homology-based transfer of binding domain annotation. The schematic shows how a catalogue of Pfam-A domains with known small molecule interactions was mapped to protein sequences in the ChEMBL target dictionary

The objective of the mapping heuristic is to annotate biological assays reported in ChEMBL with the protein domain that mediates small molecule binding. The heuristic is based on protein domain annotations provided through the Pfam-A collection of sequence-based protein domains (Punta *et al*., 2012). As a first step in the heuristic, a catalogue of Pfam-A domains capable of small molecule binding was constructed from small molecule bioactivities measured against single domain proteins from ChEMBL. PPDMs offers a facility to refine the original catalogue by adding Pfam-A domains that are known from other sources to interact with small molecules but which are missing from the catalogue in the original implementation. *Vice-versa*, Pfam-A domains can be removed from the catalogue if evidence for small molecule binding is deemed insufficient. For example, we adjusted the previously applied potency threshold of 50 µM to a more stringent threshold of 10 µM, corresponding to a pChEMBL value of 5, where pChEMBL is defined as −log_10_(molar IC50, XC50, EC50, AC50, Ki, Kd or Potency), see Bento *et al.* (2014). As a consequence, we removed a number of domains associated with weak and potentially non-specific binding. The catalogue and associated evidence for small molecule binding can be reviewed in the ‘Evidence’ section of the PPDMs resource. In a second step, this catalogue was mapped to proteins that are defined as targets in binding or functional assays where the target is either a single protein or a protein complex (defined through a relationship of type ‘D’) and a pChEMBL value is assigned. This resulted in three possible categories of outcomes (see also Fig. 1):

- A successful mapping if exactly one of the Pfam-A domain models from the catalogue matches the sequence.
- No mapping if none of the Pfam-A domain models from the catalogue match the sequence;
- A conflicting mapping if multiple domain models from the catalogue match the sequence.

Table 1 summarizes the distribution of measured activities across these three categories. Despite their relatively small contribution to the total of measured activities, protein architectures associated with category iii-type outcomes form a subset of high relevance to drug discovery, for example, many tyrosine kinases and ligand-gated ion channels. In the ‘Conflicts’ section, PPDMs provides a facility to manually assign mappings for such architectures on a per-assay basis. For each assay, PPDMs provides an overview of the assay details, domain architecture of the associated target and a form to submit a manual assignment. Assignments can be reviewed in the ‘Logs’ section, with an option of revoking a previous curation decision. User profiles ensure that accidental or deliberate assignment errors can be rolled back on a per-user basis if necessary.

**Table 1.** The table below summarizes how activities in the current release distribute over the three possible outcomes

Outcome	# All	% All	# Active	% Active
i) Successful map	750 653	53.5	269 128	76.2
ii) Not mapped	625 135	44.5	63 010	17.9
iii) Conflicting map	28 327	2.0	20 839	5.9
Total	1 404 115	100	352 977	100

Columns headed ‘all’ represent all activities, whereas columns headed ‘active’ represent activities from binding assays where pChEMBL is >5. %, percentage relative to total.

^a^Total count.

## 3 Integration with the ChEMBL database

The PPDMs workflow is decoupled from the release cycle of the ChEMBL database. Assigned mappings can be exported from PPDMs, by downloading the pfam_maps table using a link in the logs section. Equally, an up-to-date version of the catalogue (table name: valid_domains) can be downloaded from the evidence section. For integration of mappings assigned using PPDMs into the main schema of the ChEMBL database, a standardized procedure exists. Prior to each ChEMBL release, the most recent version of the catalogue is obtained from PPDMs. In a second step, it is applied to proteins that are defined as targets in assays meeting the required criteria. Finally, the set of manually assigned mappings is obtained from the PPDMs resource and used to override mappings that have been assigned by the default procedure.

## 4 Outlook

PPDMs provides a richer, domain-level perspective of small molecule binding and enriches annotation of small molecule bioactivities stored in the ChEMBL database. We anticipate that this type of annotation will improve the precision of target prediction and efficacy modelling approaches, and interpretation of the effects of natural genetic variation. PPDMs enables the refinement of domain-level annotation of small molecule bioactivities in a facile and transparent manner. The curation of conflicting mappings in PPDMs is ongoing and we are hopeful that PPDMs can engage the community in reviewing and improving domain-level annotations of small molecule bioactivities.
